# Biomonitoring of Urinary Nickel Successfully Protects Employees and Introduces Effective Interventions

**DOI:** 10.3390/ijerph19084887

**Published:** 2022-04-17

**Authors:** Che-Yu Kuo, Cheng-Fu Lin, Shih-Yu Chung, Yu-Li Lin, Wei-Min Chu, Chun-Chieh Chen, Yu-Tse Tsan

**Affiliations:** 1 Division of Occupational Medicine, Department of Emergency, Taichung Veterans General Hospital, Taichung 40705, Taiwan; s201024kobe@gmail.com (C.-Y.K.); chengfue@gmail.com (C.-F.L.); ulysses51520@hotmail.com (Y.-L.L.); williamchu0110@gmail.com (W.-M.C.); 2 Center for Geriatrics & Gerontology, Taichung Veterans General Hospital, Taichung 40705, Taiwan; 3 Division of Family Medicine, Department of Internal Medicine, Taichung Veterans General Hospital Puli Branch, Nantou 54552, Taiwan; sychungn@gmail.com; 4 School of Medicine, National Yang Ming Chiao Tung University, Taipei 11221, Taiwan; 5 School of Medicine, Chung Shan Medical University, Taichung 40201, Taiwan; sp954455@yahoo.com.tw; 6 Department of Family Medicine, Taichung Veterans General Hospital, Taichung 40705, Taiwan; 7 Department of Post-Baccalaureate Medicine, College of Medicine, National Chung Hsing University, Taichung 40227, Taiwan; 8 Institute of Health Policy and Management, National Taiwan University, Taipei 10617, Taiwan; 9 Department of Family and Community Medicine, Chung Shan Medical University Hospital, Taichung 40201, Taiwan

**Keywords:** nickel toxicity, urinary nickel, biomonitoring, workplace monitoring, behavioral change

## Abstract

Nickel is a heavy metal used in many industries. Nickel exposure can induce respiratory diseases and allergic reactions, and increase cancer risk. This study evaluated the introduction of a grinding and polishing system to prevent injuries from nickel toxicity in workers. We performed a controlled, interventional, before-and-after study from January 2018 to December 2019 at a faucet component industrial manufacturing site. Results from workplace environmental monitoring, questionnaire responses, and biomonitoring were collected before and after the intervention. Thirty-seven workers (100% men) aged 25.0 (interquartile range (IQR): 22.0–33.5) years were categorized into two groups, those with and without nickel exposure. In the exposed group, the median exposure time was 18.0 months (IQR 14.0–20.0 months). Urinary nickel concentration was lower in the exposed group than in the non-exposed group (13.8 (IQR 1.7–20.7); 23.1 (IQR 11.3–32.8) μg/g creatinine, respectively; *p* = 0.047). The median urinary nickel concentration was lower in the second year than in the first year (17.4 (IQR 2.2–27.4), 7.7 (IQR 4.3–18.5) μg/g creatinine, respectively; *p* = 0.022). Significant reductions in urinary nickel concentration were observed following the intervention and educational program. Thus, biomonitoring of urinary nickel concentration can successfully reflect the effectiveness of interventions and their relationship to nickel exposure.

## 1. Introduction

Nickel is a transition metal element with the chemical symbol Ni and atomic number of 28. It is the fifth most common metal element on Earth, and in nature, most nickel compounds exist in the form of oxides, sulfides, and silicates [1]. Nickel is a shining silver-white metal that is hard but very malleable and combines easily with other metals to form alloys [2]; for example, alloys with zinc, copper, iron, and chromium. Nickel can also combine with other elements, such as oxygen, chlorine, and sulfur to form nickel compounds [3]. Nickel and its compounds have no characteristic odors or tastes. Nickel is also widely used in a variety of products because of its high flexibility, high melting point, oxidation and corrosion resistance, and low price. In occupational settings, exposure to nickel and nickel compounds occurs primarily during nickel electrolyte plating, nickel cadmium battery manufacturing, coin manufacturing, kitchenware manufacturing, and steel manufacturing [4,5,6,7].

The general public is exposed to nickel through food, air, drinking water, smoking, and skin contact. Factories that use nickel compounds may release nickel into the air. The grinding operation will produce airborne fumes, and dusts and mists containing nickel compounds which are absorbed by human body through air exposure [8]. Eating foods containing nickel is the main source of nickel exposure for most people. Foods with high nickel content include chocolates, soybeans, nuts, and oats [9,10,11]. According to research in the United States, the daily intake of food containing nickel is approximately 69–162 μg [12,13,14]. Most of the nickel that is ingested through food is excreted through the gastrointestinal tract. Less than 10% of nickel in food is absorbed in the gastrointestinal tract, and the rest is excreted through urine and feces. Soluble nickel that is absorbed by inhalation has a serum half-life of 20–34 h and urine half-life of 17–39 h [15]. Soluble nickel can be quickly excreted from the body without bioaccumulation; therefore, urine nickel concentration only reflects recent soluble nickel exposure. Insoluble nickel exposure is related to particle size; the smaller the size, the faster it will be excreted in the urine, with a urine half-life of 30–53 h [16,17]. If the particle size is large, then the half-life can reach several months to several years [18].

The severity of various health hazards caused by nickel is affected by the dose and duration of exposure. Acute reactions caused by nickel include allergic or irritant contact dermatitis, nickel itching, and asthma [19,20]. In a chronic reaction, nickel-induced respiratory cancer can occur in the nasal cavity, ethmoid sinus, trachea, bronchus, and pleural [21,22,23]. Nickel compounds are human carcinogens that have been confirmed by the International Agency for Research on Cancer (IARC) (Group 1) [24]. Currently, various nickel compounds commonly found in daily life in different forms are believed carcinogenic to humans [25,26,27]. The DNA damage caused by nickel is found to be an important carcinogenic mechanism [28]. Therefore, avoiding nickel exposure at work is a necessary preventive measure.

This study used a before-and-after design to assess the factors associated with nickel toxicity. To the best of our knowledge, this is the first article on reducing nickel exposure using improvements in engineering and managerial intervention. No prior research on the practical application of these measures in the work environment can be found. We evaluated the effectiveness of a grinding and polishing system, along with an associated educational program, on faucet-component industrial manufacturing workers. This study focused on assessing the importance of engineering enhancements for the improvement of health in the workplace.

## 2. Materials and Methods

### 2.1. Study Design

**Figure 1:**
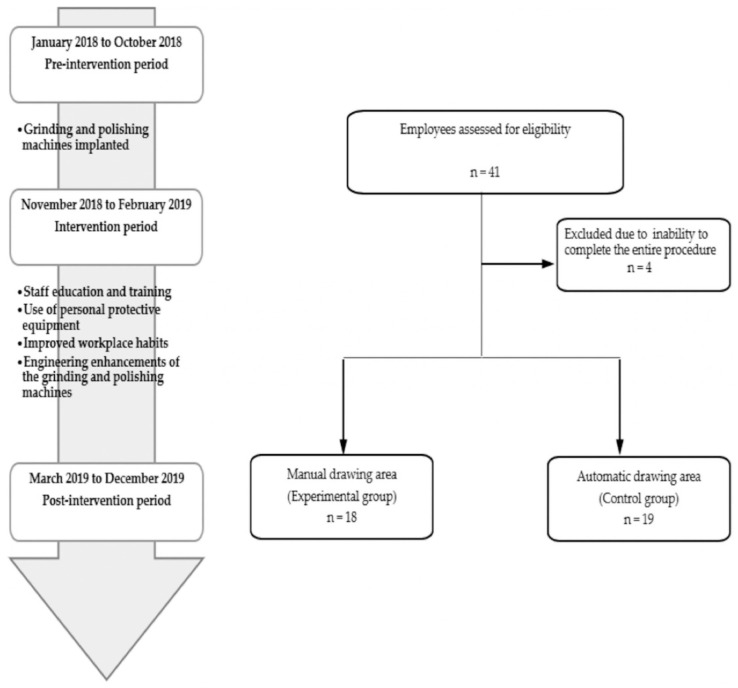
Overview of the implementation process and data collection periods.

This was a possible before-and-after study performed in a company that manufactures faucet components and produces decorative equipment for kitchens and bathrooms. The study was conducted with a hairline drawing unit divided into automatic and manual drawing areas. The employees in the manual drawing area who were exposed to nickel metal dust were regarded as the experimental group, while the others were labeled as the control group. The study design included a pre-intervention period (January 2018 to October 2018), intervention period (November 2018 to February 2019), and a post-intervention period (March 2019 to December 2019). The flow diagram is shown in Figure 1. During the pre-intervention period, grinding and polishing machines were implanted. Engineering enhancements of the grinding and polishing machines were performed, and the workers followed an associated training course in the intervention period. The training course consisted of the use of personal protective equipment and the promotion of improved workplace habits. During the post-intervention period, the manufacturing standard operating procedure was an all-round implementation that was checked by the administrative management team. This study was approved by the Institutional Review Board (IRB) of Taichung Veterans General Hospital (IRB No. CE18353A). All methods were performed in accordance with relevant guidelines and regulations. Informed consent was obtained from all subjects involved in the study.

### 2.2. Participants

This study targeted employees of the hairline drawing unit of a faucet manufacturing company. The exclusion criterion was incomplete or interrupted follow-up, leading to missing data at the end of the study.

### 2.3. Intervention

At the onset of this study, we performed a workplace evaluation to check for any possible exposure sources. Subsequently, we conducted several interventions, including administrative management, engineering enhancements of the grinding and polishing system, staff education, and training in every month from November 2018 to February 2019.

The grinding and polishing machines were in a confined space; inside the machines were self-grinding polishing wheels. When grinding, the entire confined space of the machine will reach negative pressure through the exhaust device, minimizing exposure to nickel dust in the workplace as much as possible by the exhaust device [29].

In addition to personal protective equipment recommendations involving N95 respiratory masks and Class C protective clothing, we also supplied air respirators, self-contained breathing apparatuses, and goggles. We educated workers on the importance of using personal protective equipment including how to properly put on and take off personal protective equipment (Class C protective clothing) to avoid contact with nickel dust attached to the surface of the personal protective equipment, wash their hands and face immediately after removal of the protective equipment, and prohibiting eating and drinking in the work area. Employees were asked to change into clean clothing before leaving the company to avoid bringing home nickel dust. We required employees to wear N95 respiratory masks during work and performed a fit test of mask to ensure the protective effect of the mask.

### 2.4. Data Collection

Ten months before and after the intervention, a structured interview questionnaire was administered to obtain information including personal habits, medical history, and work environment awareness and protection. The questionnaire construction referred to related study of nickel [30]. The section involving basic information included age, sex, body mass index, weight, educational level, height, and seniority of the employee. The section on personal habits included frequency of smoking, drinking, betel nut chewing, and intake of high-nickel foods (such as oats, oatmeal, chocolate, nuts, and beans). The section regarding past medical history included skin rash, allergic rhinitis, sinusitis, asthma, chronic bronchitis, and cancer. The section on working environment awareness and protection included nickel hazard awareness, personal protective equipment, and behavioral measures. Simultaneously, biomonitoring of urinary nickel and blood creatinine was performed. Moreover, nickel exposure in workplace air was assessed through workplace environmental monitoring.

### 2.5. Statistical Analyses

Continuous variables were expressed as median and interquartile range (IQR, 25–75%).

Categorical data were expressed as the number and percentage of the total number of participants. Paired comparisons were made using the Wilcoxon signed-rank test or Friedman test for continuous variables and McNemar’s or Cochran’s Q test for categorical variables. Univariate and multivariate regression analyses were used to assess the relationships between post-intervention urinary nickel concentration and demographic and clinical data. Statistical analyses were performed using SPSS version 22.0 (IBM Corp., Armonk, NY, USA). Statistical significance was set at *p* < 0.05.

## 3. Results

During the recruitment period, 41 employees were reviewed for eligibility, and 4 employees were excluded due to incomplete procedures. This resulted in 37 participants being enrolled; their characteristics are shown in Table 1.

All participants were male, with a median age of 25.0 IQR 22.0–33.5) years. The majority (54.1%) had achieved a senior high school level of education and had worked for the company for an average of 18.0 (IQR 14.0–20.0) months. Four (10.8%) participants reported smoking and 15 (40.5%) reported drinking alcohol, whereas none had a history of betel nut chewing. Nine (24.3%) participants consumed oatmeal, eight (21.6%) consumed chocolate and nuts, and six (16.2%) consumed beans. The time-weighted average of nickel in the air samples was within the permissible exposure limit. The concentration of nickel in urine at the preintervention period was 17.4 μg/g creatinine (IQR 2.2–27.4 μg/g creatinine) in 2018, and dropped significantly to 7.7 μg/g creatinine (IQR 4.3–18.5 μg/g creatinine) in 2019.

As shown in Table 2, 18 (48.6%) employees who worked in the manual drawing area, exposed to nickel metal dust, were in the experimental group, and 19 (51.4%) employees in the automatic drawing area were in the control group.

There were no significant differences between the two working areas in terms of blood creatinine, age, body mass index, marital status, education, smoking habits, and consumption of high nickel foods. However, the median urine nickel concentration in the manual drawing area was 13.8 μg/g creatinine (IQR 1.7–20.7 μg/g creatinine), which was significantly (*p* = 0.047) lower than that seen in the automatic drawing area.

The analysis of the relationship between the post-intervention period of urine nickel and other variables is shown in Table 3.

The associated training course consisted of the use of personal protective equipment and the promotion of good workplace habits, such as wearing protective clothing. The pre-intervention period of urine nickel was related to the post-intervention period of urine nickel. Moreover, in subgroup analysis of the biomonitoring results of urinary nickel (data not shown), the median urine nickel concentration before and after intervention in the automatic drawing area was 23.1 μg/g creatinine (IQR 11.3–32.8 μg/g creatinine) and 18.2 μg/g creatinine (IQR 8.8–35.0 μg/g creatinine), respectively, with no statistically significant difference (*p* = 0.398). In the manual drawing area, results showed 13.8 μg/g creatinine (IQR 1.7–20.7 μg/g creatinine) and 4.9 μg/g creatinine (IQR 2.5–5.9 μg/g creatinine), respectively, with significant change (*p* =0.014) being seen while conducting several interventions.

## 4. Discussion

In the present study, we demonstrated that the use of grinding and polishing machines, along with associated educational programs, significantly reduced nickel exposure in the workplace. In addition, urinary nickel concentration was a good biomonitoring indicator that successfully reflected employee behavioral changes. Nickel hazards can be clearly depicted through biomonitoring, and this information can be assessed by nickel-related operators. Implementation of the educational programs used in this study could help to reduce exposure to hazardous substances in the workplace. The results of the article only present an improvement in the overall urinary nickel concentration. It does not present individualized effects of managerial actions and engineering improvement. However, it still can be seen that the effectiveness of monitoring plays an important role.

This finding differed from our expectation that the median urine nickel concentration before and after intervention in the automatic drawing area was higher than that in the manual drawing area. According to a previous study, differences in personal hygiene habits are related to the concentration of exposure to chemical substances [31]. We found less employees changed their clothes before going home during off-duty in the automatic drawing area (26.3%) compared to those in the manual drawing area (55.6%); although there was no statistical significance, this could be related to the small sample size. The primary route of exposure to nickel is inhalation, and skin contact is another possible route. In this study, exposure via skin absorption was considered a limitation.

As shown in Table 2, there were no differences between exposure and control group in non-occupational exposure factors such as alcohol drinking, smoking, high nickel food intake habit (oatmeal, chocolate, nuts, beans), and personal hygienic habits. Intake of high-nickel foods and smoking habits were not correlated with nickel concentration in the urine, and our study supported the finding that it was less likely for people to be affected by non-occupational nickel exposure. During the entire study period, the environmental detection of nickel was not significantly different, thus indicating that workers continued to be exposed to nickel throughout the study period. Furthermore, based on observations and discussions with supervisors, the company’s production capacity and the work process of the complete project did not change during the study period. This is more indicative of the correlation between nickel concentration in urine and work.

There are great benefits to using educational methods to reduce employee exposure to toxic chemicals. Due to economic considerations, not all companies can implement engineering improvements, but education methods can have a lasting effect on personal hygiene through educational actions and has low cost. The company strengthened its work regulations by offering an educational program, which included teaching the importance of washing face and hands prior to eating during on-duty hours and wearing personal protective equipment while working, which can significantly improve employee safety education knowledge [32]. This finding was similar to that of many other studies, which showed that employees’ personal working habits and personal hygiene can cause differences in exposure [8,33,34]. Our study also found that it is necessary to educate employees regarding the importance of using personal protective equipment as well as outlining necessary guidelines for explaining how properly putting on and taking off work clothes could reduce exposure to nickel. The univariate linear regression analysis (see Table 3) revealed wearing protective clothing and changing clothes showed significant relationships between urinary nickel post-intervention. Since occupational hazards related to nickel have widely been reported in recent years, utilizing the educational approach in this study can be an effective method to reduce potential exposure in workplaces.

Nickel concentrations in the blood and urine are the main tools for assessing exposure to insoluble nickel compounds and are widely used as indicators of nickel exposure [35,36]. However, some studies argue differently because nickel concentrations in urine and blood may not completely reflect actual exposure concentration [37]. As insoluble nickel compounds can accumulate in the lungs, the time at which the compounds can be absorbed into the blood and excreted via urine changes. However, our study results were consistent with the conclusion that by analyzing urine nickel, behavioral changes could be detected, while environmental detection of nickel was under the permissible exposure limit-time weighted average.

This study had certain limitations. First, our study had a small sample size, and the study data were collected over a period of only 2 years. It is hoped that future research can be applied to a larger sample size and a combination of different workplaces can be evaluated. In addition, no longer-term follow-up has been completed to assess how changes in worker behaviors have been maintained. Future studies may consider relevant analyses. Second, the questionnaire was completed in a self-reported manner; therefore, recall bias may have occurred. Third, spot urine was collected to measure nickel concentration when it was preferable to collect 24 h urine samples for more accurate monitoring. However, collecting 24 h urine samples is impractical in an occupational setting.

## 5. Conclusions

By conducting three interventions including improving the ventilation exhaust system of the grinding and polishing workplace, staff education, and personal protective equipment recommendations, significant benefits to employees away from nickel hazards can be achieved. The awareness of these interventions comes from the biomonitoring of urine nickel, as an indicator of nickel exposure. Therefore, to generate the will to act in accordance with the procedure is important, and self-monitoring can be a tool to help to achieve this aim. Healthcare managers can make good use of biomonitors to achieve healthy workplace goals.

## Figures and Tables

**Table 1:** Baseline characteristics of the participants.

	Total (*n* = 37)	
Demographic characteristics	Median	(IQR)
Age (years)	25.0	(22.0–33.5)
Height (cm)	168.0	(164.0–173.0)
Weight (kg)	61.0	(53.5–68.0)
Body mass index (kg/m^2^)	21.2	(19.2–23.5)
Seniority (months)	18.0	(14.0–20.0)
Marital status		
Single	23	62.2%
Married	13	35.1%
Widowed	1	2.7%
Educational level		
Junior high school	2	5.4%
Senior high school	20	54.1%
Junior college	6	16.2%
University	9	24.3%
Smoking status		
Never/Former	33	89.2%
Current	4	10.8%
Alcohol		
No	22	59.5%
Yes	15	40.5%
Intake oatmeal	9	24.3%
Intake chocolate, nuts	8	21.6%
Intake beans	6	16.2%
Hygienic program education		
During working hours		
Wearing masks	35	94.6%
Wearing gloves	35	94.6%
Wearing protective clothing	12	32.4%
Before eating during on-duty hours		
Washing hands	35	94.6%
Before going home during off-duty		
Changing clothes	15	40.5%
Past history		
Sinusitis	2	5.4%
Skin rashes	1	2.7%
Health inspection report		
2018 Urinary nickel (μg/g creatinine)	17.4	(2.2–27.4)
2019 Urinary nickel (μg/g creatinine)	7.7	(4.3–18.5)
Blood creatinine (mg/dL)	0.8	(0.8–0.9)

IQR, interquartile range.

**Table 2:** Characteristics of participants according to exposure levels to nickel.

	Manual Drawing Exposed Group (*n* =18)		Automatic Drawing Non-Exposed Group (*n* = 19)		*p* Value
Demographic characteristics					
Age (years)	25.0	(22.8–33.3)	26.0	(22.0–34.0)	0.851
Height (cm)	166.0	(162.8–169.3)	172.0	(167.0–178.0)	0.014 *
Weight (kg)	59.0	(51.0–64.3)	65.0	(56.0–75.0)	0.048 *
Body mass index (kg/m^2^)	21.3	(19.1–22.7)	21.2	(19.1–25.6)	0.412
Seniority (months)	14.0	(5.0–18.0)	19.0	(18.0–21.0)	0.004 *
Marital status					0.579
Single	11	61.1%	12	63.2%	
Married	7	38.9%	6	31.6%	
Widowed	0	0%	1	5.3%	
Educational level					0.065
Junior high school	1	5.6%	1	5.3%	
Senior high school	13	72.2%	7	36.8%	
Junior college	3	16.7%	3	15.8%	
University	1	5.6%	8	42.1%	
Smoking status					1.000
Never/Former	16	88.9%	17	89.5%	
Current	2	11.1%	2	10.5%	
Alcohol					0.032 *
No	7	38.9%	15	78.9%	
Yes	11	61.1%	4	21.1%	
Intake oatmeal	3	16.7%	6	31.6%	0.447
Intake Chocolate, Nuts	3	16.7%	5	26.3%	0.693
Intake beans	2	11.1%	4	21.1%	0.660
Hygienic program education					
During working hours					
Wearing masks	16	88.9%	19	100%	0.230
Wearing gloves	16	88.9%	19	100%	0.230
Wearing protective clothing	7	38.9%	5	26.3%	0.642
Before eating during on-duty hours					
Washing hands	16	88.9%	19	100%	0.230
Before going home during off-duty					
Changing clothes	10	55.6%	5	26.3%	0.140
Past history					
Sinusitis	1	5.6%	1	5.3%	1.000
Skin rashes	0	0%	1	5.3%	1.000
Health inspection report					
2018 Urinary nickel (μg/g creatinine)	13.8	(1.7–20.7)	23.1	(11.3–32.8)	0.046 *
2019 Urinary nickel (μg/g creatinine)	4.9	(2.5–5.9)	18.2	(8.8–35.0)	0.000 *
Blood creatinine (mg/dL)	0.8	(0.8–0.9)	0.8	(0.8–0.9)	0.201

* *p* < 0.05.

**Table 3:** Relationships between post-intervention urinary nickel levels and demographic and clinical data.

	Univariate Linear Regression			Multivariate Linear Regression		
	B	Beta (β)	*p* Value	B	Beta (β)	*p* Value
Unit	−17.402	−0.597	0.000 *	−10.851	−0.372	0.004 *
Height (cm)	0.935	0.391	0.017 *	0.044	0.019	0.905
Weight (kg)	0.478	0.397	0.015 *	0.053	0.044	0.750
Seniority (months)	0.962	0.408	0.012 *	0.009	0.004	0.978
Alcohol	−12.945	−0.437	0.007 *	−2.602	−0.088	0.484
Wearing protective clothing	−10.331	−0.332	0.045 *	−0.596	−0.019	0.884
Changing clothes	−12.710	−0.429	0.008 *	−6.747	−0.228	0.126
Skin rashes	47.794	0.532	0.001 *	40.686	0.453	0.000 *
2018 Urinary nickel (μg/g creatinine)	0.383	0.517	0.001 *	0.285	0.386	0.008 *
Educational level	5.241	0.329	0.047 *	−4.046	−0.254	0.129
Age (years)	0.594	0.246	0.142			
Body mass index (kg/m^2^)	1.230	0.289	0.083			
Marital status	2.568	0.096	0.573			
Smoking status	−8.182	−0.174	0.302			
Intake oatmeal	1.193	0.035	0.836			
Intake chocolate, nuts	2.539	0.072	0.673			
Intake beans	10.049	0.254	0.129			
Washing hands	5.917	0.092	0.589			
Sinusitis	6.504	0.101	0.552			
Blood creatinine (mg/dL)	30.511	0.180	0.287			

* *p* < 0.05.

## Data Availability

Not applicable.
